# The effect of autologous adipose derived mesenchymal stem cell therapy in the treatment of a large osteochondral defect of the knee following unsuccessful surgical intervention of osteochondritis dissecans – a case study

**DOI:** 10.1186/s12891-017-1658-2

**Published:** 2017-07-14

**Authors:** Julien Freitag, Kiran Shah, James Wickham, Richard Boyd, Abi Tenen

**Affiliations:** 1 Charles Sturt University, NSW, Australia; 2 Magellan Stem Cells, VIC, Australia; 3 The Hudson Institute, Victoria, Australia; 4 Monash University, VIC, Australia

**Keywords:** Osteochondritis dissecans, Osteochondral defect, Osteoarthritis, Arthritis, Mesenchymal stem cells, Case study

## Abstract

**Background:**

A prospective analysis of the effect of autologous adipose derived mesenchymal stem cell (MSC) therapy in the treatment of an osteochondral defect of the knee with early progressive osteoarthritis following unsuccessful surgical intervention of osteochondritis dissecans (OCD).

**Case presentation:**

After failed conventional management of OCD a patient undergoes intra-articular MSC therapy. Patient outcome measures included the Numeric Pain Rating Scale (NPRS), the Western Ontario and McMaster Universities Arthritis Index (WOMAC) and the Knee Injury and Osteoarthritis Outcome Score (KOOS). Structural outcome was assessed using MRI with the novel technique of T2 mapping used to indicate cartilage quality. Following MSC therapy the patient reported improvement in pain and function as measured by NPRS, WOMAC and KOOS. Repeat MRI analysis showed regeneration of cartilage. MRI T2 mapping indicated hyaline like cartilage regrowth.

**Conclusion:**

In this report, the use of MSCs, after unsuccessful conventional OCD management, resulted in structural, functional and pain improvement. These results highlight the need to further study the regenerative potential of MSC therapy.

**Trial registration:**

Australian and New Zealand Clinical Trial Registry Number - ACTRN12615000258550 (Date registered 19/03/2015 – retrospectively registered).

## Background

Osteochondritis dissecans (OCD) was a term first used to describe the development of loose bodies within a joint with no prior evidence of significant trauma [1]. Whilst the pathophysiology of OCD remains uncertain, it is accepted that it is a subchondral lesion characterized by variable degrees of bone resorption, collapse, sequestration and overlying articular cartilage disruption [2, 3].

Commonly held belief is that OCD may result from inflammation, vascular deficiency, genetic predisposition or repetitive microtrauma. Interestingly, OCD was initially termed osteochondritis due to a belief that it resulted from an inflammatory process [4]. Histological studies have, however, demonstrated areas of necrosis within OCD lesions rather than an inflammatory process. Repetitive microtrauma has become an accepted preceding stimulus due to the observed increased incidence of OCD amongst the athletic population [5–7].

OCD typically presents as activity related knee pain without prior history of trauma [8, 9]. Whilst it may affect several joints it is commonly described in the knee where it is most commonly seen in the lateral aspect of the medial femoral condyle (51%). Other areas of the knee include the central medial femoral condyle (19%), lateral femoral condyle (17%), medial edge of the medial femoral condyle (7%) and the patella (7%), with up to 20% of lesions being bilateral [7, 10, 11].

OCD lesions are often described as stable or unstable with stable lesions considered to be suitable for non-operative conservative management [7, 12]. De Smet and colleagues, using a T2 weighted MRI technique, noted four criteria with an observed correlation with instability – 1) a high signal line beneath the lesion, 2) a focal area of overlying articular cartilage defect, 3) a fracture of the articular cartilage, 4) subchondral cyst formation [13, 14]. Using these criteria Kijowski and colleagues showed that if all four criteria are present this correlated with a high surgical observation of instability in adult OCD, though surprisingly this had low sensitivity in predicting instability in childhood OCD [15].

It is generally accepted that first line treatment for radiologically stable OCD lesions in children should consist of a non-operative management strategy [16, 17]. Protocols for non-operative management are however inconsistent and may include non-weight bearing, casting, protected weight bearing, bracing and / or activity modification. Rates of radiological healing with non-operative management have been reported to vary from 50 to 94% [18].

Surgical management of stable lesions that fail conservative management can include drilling, bone grafting and fixation [18–20]. Whilst arthroscopic drilling has become a commonly accepted practice and has a healing response of 82–98%, extra-articular trans-epiphyseal drilling has been proposed as an alternative as it does not violate the overlying intact articular cartilage [21–23].

Surgical fixation of unstable lesions may include curettage and drilling of the underlying bone, bone grafting if necessary and fixation of the OCD using compression screws or bio-absorbable implant/nails [24]. In the case where the OCD lesion is fragmented and unable to undergo surgical fixation then the lesion/fragment should be removed. Whilst removal of the loose body may result in short term pain improvement, up to 79% of patients will have degenerative findings on plain film at 11 years follow-up [25]. This is in keeping with observed progression of osteochondral defects to osteoarthritis [26, 27]. The observed progressive joint degeneration has seen the exploration of additional interventions to promote healing.

Whilst microfracture remains a technique commonly used for osteochondral defects, its use in OCD is less well defined and outcome may arguably be influenced by the pathology of the subchondral bone that is observed in OCD. A randomised study comparing microfracture to osteochondral autologous transplantation in OCD demonstrated similar short-term results. The microfracture group, however, deteriorated with time and exhibited a failure rate of 41% at 4 years post operatively. Research by Britterg and colleagues, using autologous chondrocyte implantation, has shown excellent outcome in the management of chondral defects with success seen in osteochondral lesions as deep as 9 mm [28].

The observed progression of osteochondral defects to early degenerative osteoarthritis poses a particular problem with patients with OCD who fail surgical fixation or alternative treatment of the defect following removal of an unstable fragment. The role of mesenchymal stem cells (MSCs), with their inherent ability to differentiate along different cell lines of mesodermal lineages (including osteoblasts and chondrocytes) coupled with their observed paracrine expression of several growth factors and cytokines which promote tissue repair, has seen a renewed focus on their potential in orthopaedic regenerative techniques [29–32]. Within both pre-clinical and clinical literature there is a growing indication of the possible efficacy of MSC therapy in the management of osteochondral defects and similarly in osteoarthritis.

Preclinical trials using techniques similar to autologous chondrocyte transplantation but replacing chondrocytes with MSCs have shown positive outcomes with noted formation of hyaline like cartilage at the site of repair [33, 34]. Wakitani and colleagues have used this similar technique in a limited clinical trial to successfully treat isolated cartilage defects [35].

The use of microdrilling (a technique similar to microfracture) in combination with intra-articular MSC injections in the treatment of osteochondral defects has shown initial promise with later biopsy showing histopathology consistent with type II collagen and hyaline like cartilage formation [36]. The authors of this case report are also involved in a concurrent randomised controlled trial assessing a similar microfracture technique in combination with autologous adipose derived stem cells [37].

Whether stem cell therapies have similar observable benefits in the treatment of osteochondritis dissecans is yet to be formally studied.

## Case presentation

### Medical history and assessment

A 26 year old male presented with increasing right knee pain and functional debility with a history of osteochondritis dissecans and having had multiple past surgical interventions. He was otherwise well, though was currently unable to perform any activities that involved prolonged weight bearing and found it increasingly difficult to continue his occupation within allied health care.

The patient noted an initial diagnosis of OCD at age 13. This was appropriately treated conservatively with a period of reduced load and protected weight bearing. He was later able to return to sport - which included Australian Rules football.

Due to recurrence of pain he underwent arthroscopic examination at age 14. Arthroscopic probing of the OCD lesion indicated that it was stable and conservative management and further unloading was pursued. The patient failed conservative management and 1 year later underwent a repeat arthroscopy at which time an unstable 3 cm × 3 cm lesion involving his medial femoral condyle was removed. It was felt that as the fragment had little subchondral bone that direct repair was not achievable. A further arthroscopy was performed 8 months later and a chondral biopsy was taken for later autologous chondrocyte transplantation. At the time it was noted that no healing had occurred at the site of the defect.

Two months later, the treating surgeon, using a lateral arthrotomy approach, patched the defect using a matrix induced autologous chondrocyte implant (MACI). The patch was fixed in place using fibrin glue. One year later, and due to persistent discomfort and swelling of the knee, the patient underwent a further arthroscopy. It was noted that the outer rim of the MACI patch had incorporated well, yet an inner area of 1.5 cm × 1.5 cm had failed to fill completely.

At age 23 and due to troubling discomfort and mechanical features of instability the patient underwent another arthroscopy. Numerous loose bodies within both the medial and lateral compartments were washed out. The area of the past OCD and MACI graft had failed with exposure of underlying subchondral bone and formation of a large subchondral cyst. Unstable edges of the area of MACI graft were debrided but as the entrance to the subchondral cyst was small a surgical decision was made not to further expose nor debride the cavity.

Failing to symptomatically improve, the patient underwent a further arthrotomy with debridement of the subchondral cyst, after which it was filled with bone graft substitute cortico-cancellous crunch granules. A periosteal flap taken from the medial border of the tibia was then applied over the lesion. The patient underwent a final arthroscopy at age 25 for debridement of an unstable fibro-cartilaginous cap at the site of the past periosteal flap.

In total the patient underwent seven separate operations.

Initial examination upon presentation showed evidence of a moderate right knee effusion. The patient had full range of motion and the knee was stable.

**Fig. 1:**
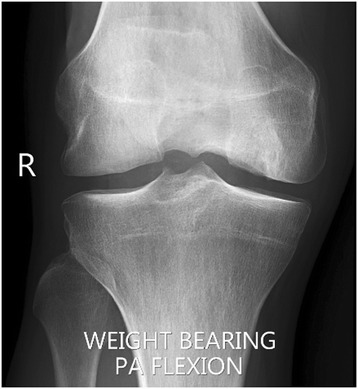
X-ray of the knee showing flattening of the medial femoral condyle and intercondylar notch osteophyte formation – Kellgren Lawrence Grade II

**Fig. 2:**
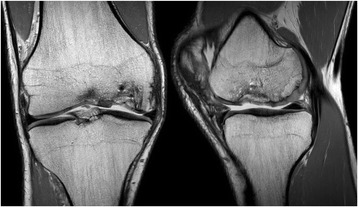
Pre-Treatment Proton Density (PD) weighted Coronal and Sagittal MRI images of the knee showing the isolated chondral defect involving the central weight bearing area of the medial femoral condyle

**Fig. 3:**
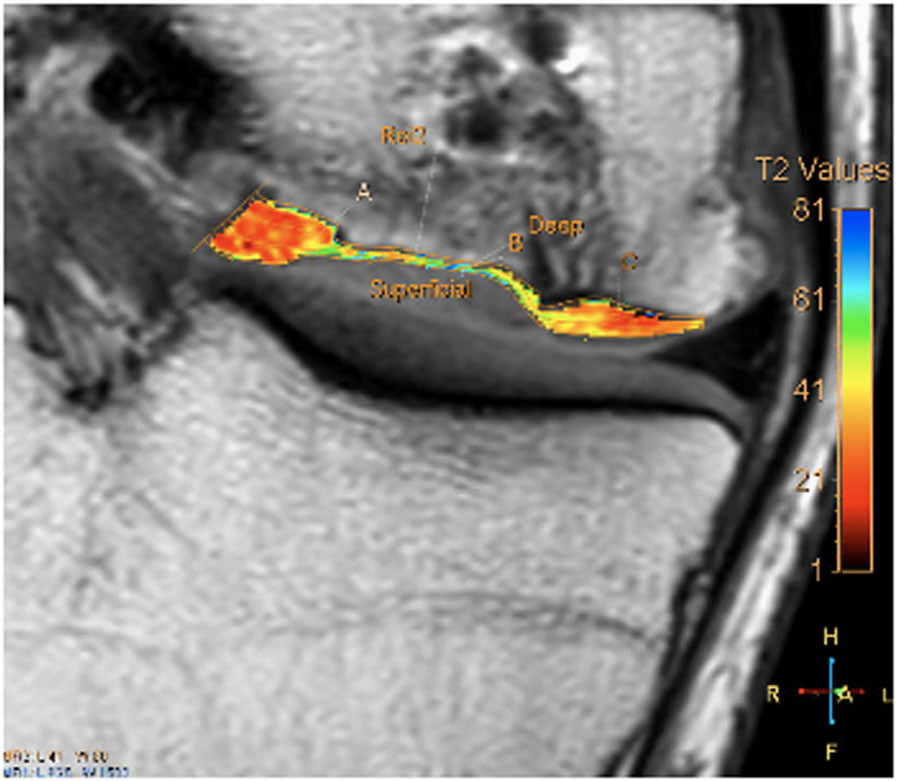
Pre-Treatment MRI T2 mapping of the medial compartment of the knee indicating elevated hydration of the cartilage in the area surrounding the OCD and also of the thin layer of tissue which partially covered the defect

Radiological examination included both X-Ray and MRI. X-Ray confirmed early degenerative change with intercondylar notch osteophytes consistent with Kellgren-Lawrence Grade II. There was noted concavity to the lateral aspect of the medial femoral condyle consistent with the previous area of OCD (Fig. 1). MRI showed marked abnormality over the weight bearing portion of the medial femoral condyle measuring 2.3 cm × 1.5 cm. The subchondral cyst had successfully been debrided and grafted and was no longer present on MRI. A thin layer of hyper-intense tissue incompletely covered the area of abnormal cortex (at site of past bone substitute grafting) (Fig. 2). The area of pathology had a modified International Cartilage Repair Society (ICRS) score of 4. In addition to routine MRI protocols, the method of T2-relaxation time cartilage mapping was used. T2 mapping indicated significantly elevated values within the thin layer overlying the osteochondral defect but also elevated values within the surrounding cartilage (Fig. 3).

The patient sought advice regarding further interventions that may improve both his current symptoms but also his long-term prognosis as he wished to avoid progressive degeneration and early joint arthroplasty. After careful consideration and as he now had features of early degenerative change he was assessed as suitable for inclusion in an ethics approved, registered case series on the use of adipose derived MSC therapy in the treatment of osteoarthritis.

The patient was given written information regarding the use of MSC therapy, including relative risks of MSC therapy and also relevant treatment alternatives that could otherwise be explored. Formal written informed consent was obtained prior to commencing therapy.

### Source of mesenchymal stem cells

Adipose tissue was chosen as a source of MSCs due to ease of harvest, abundance of MSCs and the observed chondrogenic potential of adipose derived MSCs. Bone marrow aspirate was considered an alternative source or MSCs but surprisingly has a relative paucity of MSCs – comprising .001–.02% of the mono-nucleated cell population in comparison to ~1–7% found within adipose tissue [38–40]. Many studies have indicated similar chondrogenic potential between bone marrow derived and adipose derived MSCs [41].

### Autologous adipose derived mesenchymal stem cell preparation

The patient underwent an abdominal liposuction harvest procedure. Using a lateral abdominal approach, the subcutaneous fat was infiltrated with an anaesthetic tumescent fluid preparation comprising of 30 mls of 2% lignocaine, 1 ml of 1:1000 adrenaline, 1 ml of 8.4% bicarbonate suspended in a normal saline solution (total 1000 ml). Using a 4 mm lipo-aspirate cannula, 60 mls of adipose tissue and tumescent fluid was successfully harvested and collected within a sterile medical grade single use Shippert Tissu-Trans Collection filter (Shippert Medical, Colorado, USA). The sample was then transferred directly from the theatre via an air lock system to a laboratory clean room facility operated by Magellan Stem Cells (Magellan Stem Cells, Melbourne, Australia).

Autologous MSCs were isolated and expanded from the harvested adipose tissue using previously published protocols [42]. The processing of the lipo-aspirate was performed within the environment of a Biological Safety Cabinet (BSC) Class II using strict sterile techniques.

Stromal Vascular Fraction (SVF) obtained from the lipo-aspirate was culture purified using standard growth media containing Minimum Essential Media Eagle (MEM) supplemented with 2 mM glutamate and 10% Fetal Bovine Serum (FBS) (HyClone – GE Healthcare, USA). The cells were cultured under hypoxic conditions with 5% CO_2_ at 37 °C. Non-adherent cells were removed by washing with sterile phosphate buffered saline (PBS). The adherent cells were cultured until 80% confluency in freshly added growth media. The cultured purified cells were then harvested at passage 0 (P0). These cells were further plated to tissue culture flasks and expanded up to passage 2 (P2). The cells were harvested and analyzed for cell count and viability using a Muse Cell Analyzer (Merck Millipore, USA). The harvested cells were washed three times to remove FBS and cryopreserved in cryovials using clinical grade qualified MSC cryoprotectant media and following a previously validated control rate freezing technique of 1 °C/min [43, 44]. The cryovials were transferred to liquid nitrogen storage until use.

**Fig. 4:**
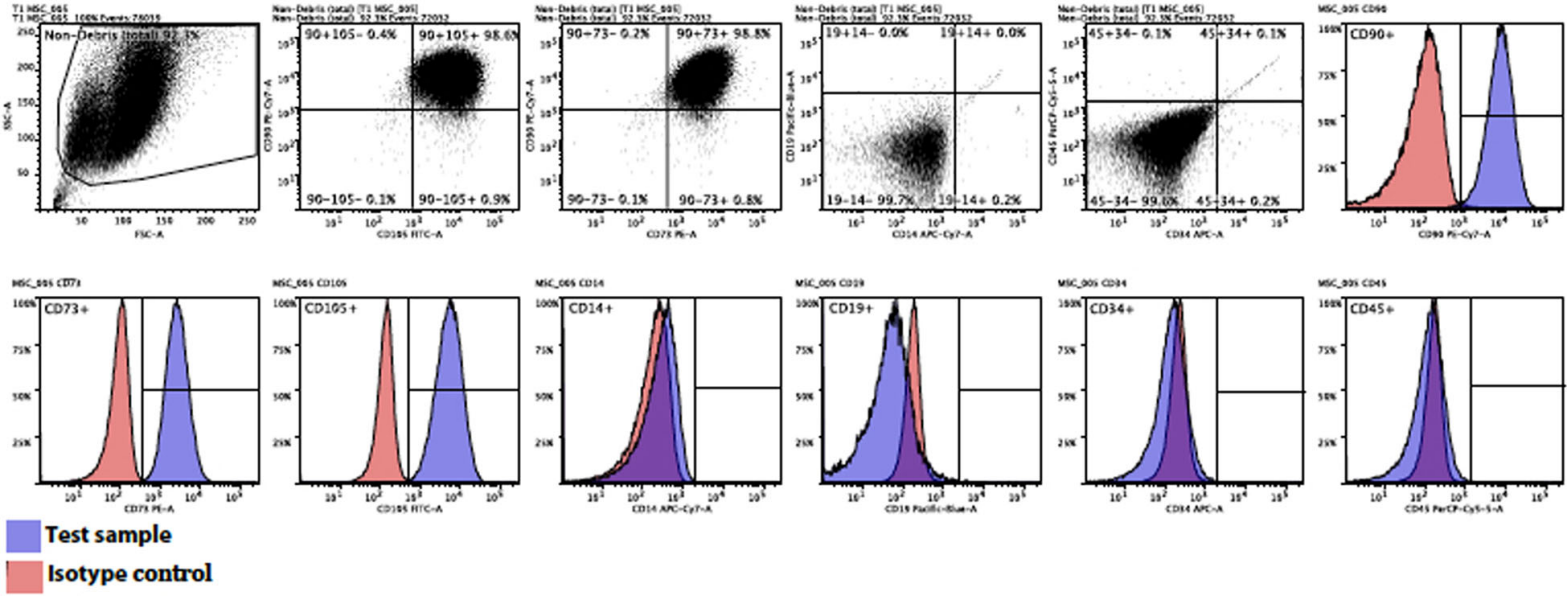
Flow Cytometry *Dot-Plot* and Histogram surface marker analysis. The *pink* and *purple* histograms represent isotope control and the tested cell sample respectively

At completion of isolation and expansion, the cells underwent independent phenotypic analysis at Monash University and were characterised by flow cytometry using Florescent Activated Cell Sorting (FACS). Using standards established by the Internal Society of Cellular Therapy, the cells were assessed for the presence of positive surface markers CD 90, CD 73 and CD 105 and absence of hematopoetic surface markers CD14, CD19, CD34 and CD45 [45] (Tables 1-2 and Fig. 4). A further sample was sent for independent sterility testing for microbial growth.

**Table 1:** Flow Cytometry Dot-Plot surface marker analysis showing results consistent with mesenchymal stem cells as per the International Society of Cellular Therapy guidelines

	Dotplots - Positive Markers		Dotplots - Negative Markers	
	CD90 & CD105 + ve	CD90 & CD73 + ve	CD14 & CD19 + ve	CD34 & CD45 + ve
Percentage	98.64	98.83	0.04	0.14

**Table 2:** Flow Cytometry Histogram surface marker analysis showing results consistent with mesenchymal stem cells as per the International Society of Cellular Therapy guidelines

	Histograms - Positive Markers			Histograms - Negative Markers			
	CD90 + ve	CD73 + ve	CD105 + ve	CD14 + ve	CD19 + ve	CD34 + ve	CD45 + ve
Percentage	99.01	99.67	99.69	0.28	0.07	0.32	0.29

### Injection method

Prior to the intra-articular injection the cryopreserved cells were retrieved from the liquid nitrogen Dewar and thawed quickly within a sterile 37 °C water bath. The thawed cells were then washed in chilled PBS to remove cryoprotectant media, centrifuged and the resultant cell pellet re-suspended in clinical grade 0.9% normal saline. The cells were again analyzed for cell count and viability post thaw using the Muse Cell Analyzer. The cells were injected within 30 min of thawing.

The patient received a total of 118 million MSCs (viability 98%) suspended in 3mls of normal saline at commencement of therapy. A second injection of 50 million MSCs (viability 95%) at 6 months was also given.

At both injection time points the patient’s right knee was prepped using a chlorhexidine solution and draped. 2 mls of 1% lignocaine was infiltrated subcutaneously at the site of injection. Using a supero-lateral approach to the patella, and under sterile conditions and ultrasound guidance, the MSCs suspended in 3 mls of normal saline were injected into the intra-articular space.

### Post injection rehabilitation

After the initial injection the patient was fitted with a medial compartment customised unloading knee brace to allow protected but full weight-bearing. This was achieved using an Ossur OA Unloader One brace (Ossur, Reykjavic, Iceland).

The patient was given post injection instructions, which included range of motion exercises and lower limb muscle activation exercises, and encouraged to perform repetitive low impact and low load exercises against minimal resistance on a stationary bike (continuous active motion versus continuous passive motion). This protocol was developed due to previously published evidence which has indicated the benefit of controlled load on cartilage health [46].

### Potential side effects / risks

Importantly, recent systematic review and meta-analysis of previous clinical papers investigating the use of mesenchymal stem cell therapy – both intravascular and intra-articular applications – and including autologous, allogeneic and expanded MSC preparations has indicated that MSC therapy is safe. No adverse events including infection, death or malignancy have been recorded.

Saw and colleagues have documented a self-limited flare up with discomfort and swelling following intra-articular MSC therapy [36]. This was not considered a serious adverse event.

Relative risks of the liposuction harvest procedure include infection, bruising and post operative discomfort. Whilst the risk of infection is low, the patient received a single dose of prophylactic antibiotics prior to the procedure as part of accepted routine clinical practice [47].

### Analysis methods and outcome measures

Prospective analysis of patient outcome to intra-articular MSC therapy included the following measures:

1. The Knee Injury and Osteoarthritis Outcome Score (KOOS). This is a validated scoring system intended for the assessment of knee injury that may result in post-traumatic knee osteoarthritis. The score consists of 5 subscales - pain, other symptoms, function in daily living, function in sport and recreation and knee related quality of life. Standardised answers to questions are given (5 Likert scale) and each question is assigned a score of 0–4. A normalized score is calculated for each subscale (100 indicates no symptoms and 0 indicates maximum symptoms) [48].
2. The Western Ontario and McMaster Universities Arthritis Index (WOMAC Index 3.0). This score is a validated quality of life score and quantitatively assesses the pain, stiffness and physical function in patients with symptomatic osteoarthritis [49].
3. The Numeric Pain Rating Scale (NPRS). The patient rates their knee pain intensity over the previous week on a scale of 0–10. The NPRS has been validated for use in people with knee osteoarthritis [50].

Outcome scores were completed at baseline, 1, 3, 6, 12 and 18 months post commencement of MSC therapy and recorded using the software program Clinical Intelligence (Clinical Intelligence, Melbourne, Australia).

Structural outcome was assessed using MRI imaging performed prior to commencement of therapy and again at 6 and 18 months post-treatment. Semi quantitative measures of the cartilage defect were obtained using a modified ICRS score [51]:

- Grade 0: normal cartilage
- Grade 1: focal blistering and intra-cartilaginous low-signal intensity area with an intact surface and bottom
- Grade 2: irregularities on the surface or bottom and loss of thickness of less than 50%
- Grade 3: deep ulceration with loss of thickness of more than 50%
- Grade 4: full-thickness cartilage wear with exposure of subchondral bone.

In addition to routine MRI protocols, the method of T2-relaxation time cartilage mapping was used. T2 mapping assesses the water content of cartilage by giving a quantifiable value to the ability of free water protons to exchange energy and move through a cartilage matrix [52]. Observed increased water content resulting in an increased T2 relaxation time is an indication of chondral pathology. T2 mapping has been well studied and is an accepted and validated non invasive measure of cartilage quality [53].

### Effect of MSC therapy on pain, function and structure

**Fig. 5:**
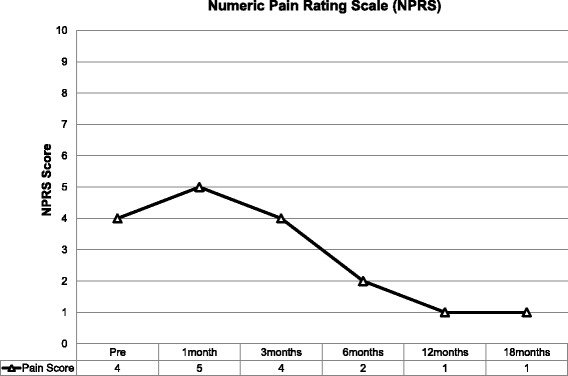
Numeric Pain Rating Score. Pain initially increased due to a self-limiting flare up following injection of MSC therapy. Thereafter it reduced and remained improved until completion of follow up at 18 months

The numeric pain rating score increased at 1 month from 4 to 5 reflecting an initial self-limiting flare up (see Adverse Events). Follow-up at 6 months however showed a 50% improvement in NPRS from baseline and this had further improved by 75% with a pain score of 1 at completion of follow-up at 18 months (Fig. 5).

**Fig. 6:**
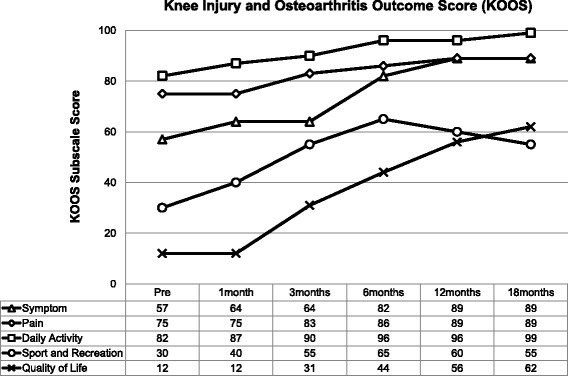
Knee Injury and Osteoarthritis Outcome Score. All subscales of KOOS showed consistent improvement over the period of follow-up

Knee Injury and Osteoarthritis Outcome Scores consistently improved across the period of follow-up (Fig. 6). At 6 months the patient’s Symptoms Score had improved by 44% and similarly his Sport and Recreational Score had improved by over 100%. These values showed continued improvement at 18 months. The measure of Quality of Life had improved at 18 months by greater than 400% above baseline.

**Fig. 7:**
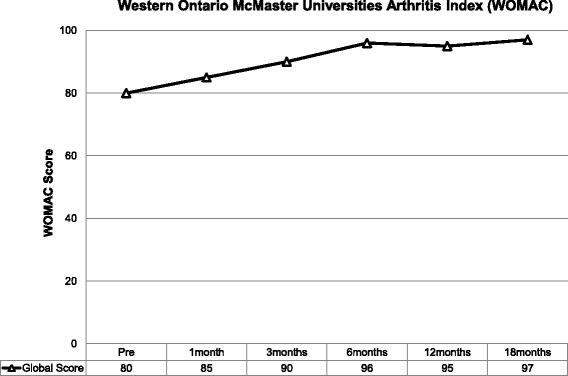
Western Ontario and McMaster Universities Arthritis Index. Pain, stiffness and function improved following mesenchymal stem cell therapy

Reflecting the observed improvement in KOOS, the WOMAC Knee Score showed similar consistent improvement from baseline till completion of data collection. At 18 months, the Global WOMAC Score had improved by over 20% (Fig. 7).

**Fig. 8:**
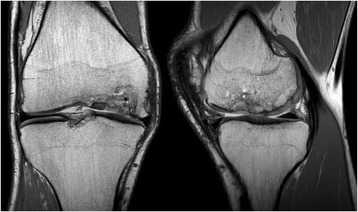
Post-Treatment PD weighted Coronal and Sagittal MRI imaging at 6 months indicating articular cartilage regeneration at the site of the osteochondral defect with smooth integration with the surrounding joint surface

**Fig. 9:**
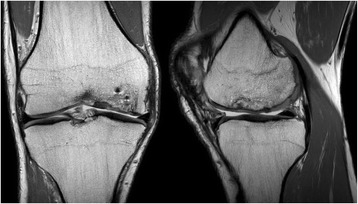
Post-Treatment PD weighted Coronal andSagittal MRI imaging at 18 months indicating persistent articular cartilage regeneration at the site of the osteochondral defect

Structural follow-up using MRI at both 6 months and 18 months showed significant increase in tissue covering the extensive OCD of the medial femoral condyle. Observed development of a subchondral plate suggested a layering effect from subchondral bone through to the chondral surface. There was smooth integration with the surrounding native cartilage at the periphery of the OCD (Figs. 8 and 9). Using the modified ICRS scoring system as described above, ICRS grade improved from 4 to 1.

**Fig. 10:**
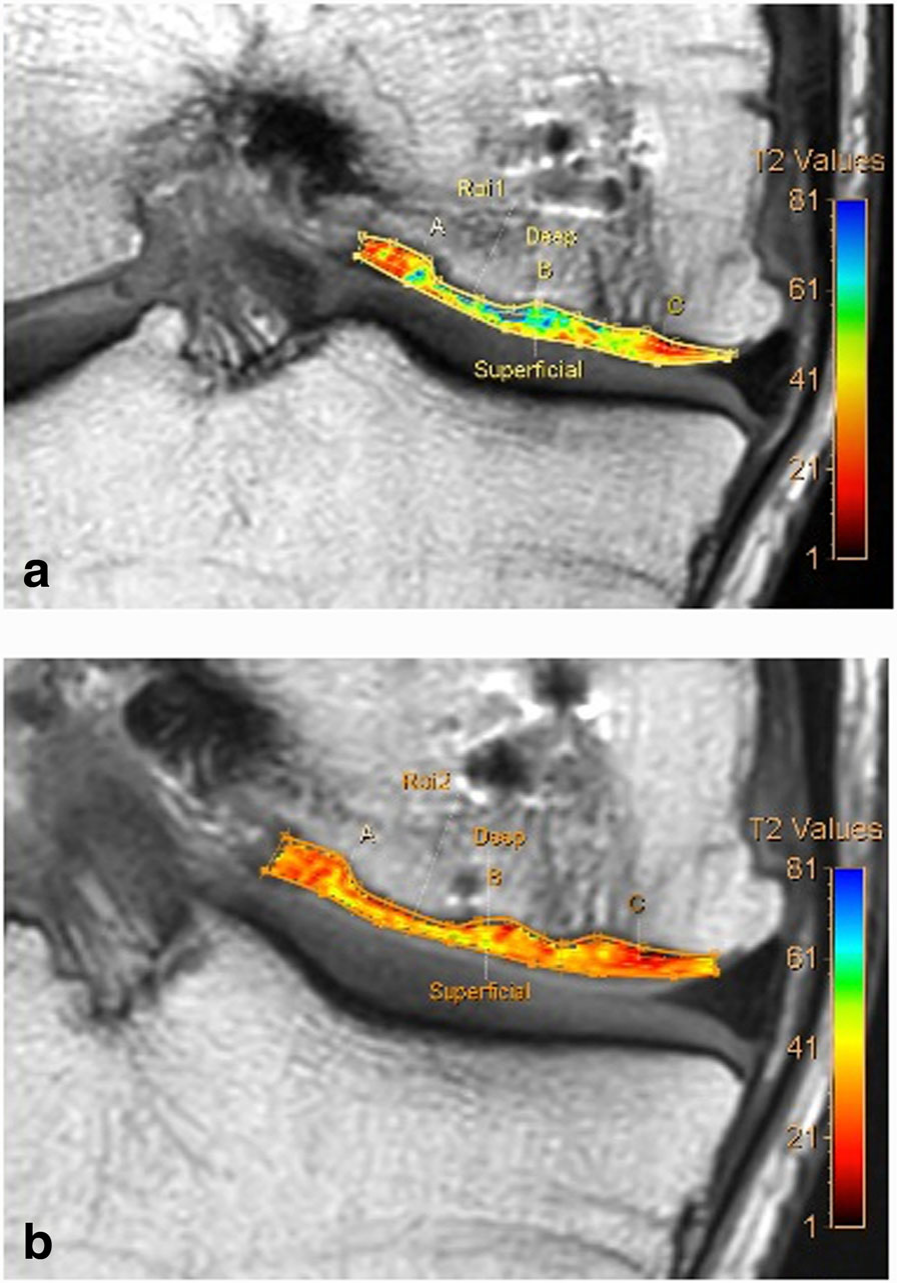
Post-Treatment MRI T2 mapping of the medial compartment of the knee. **a** 6 month T2 Mapping: Elevated values within the regenerated cartilage indicate immature cartilage or fibrocartilage morphology. **b** 18 month T2 Mapping: Improved values within both the deep and superficial layers indicate hyaline cartilage. This finding indicates cartilage maturation since previous imaging at 6 months

T2 mapping at 6 months returned elevated values at the site of regenerative cartilage, consistent with high water content and immature cartilage (Fig. 10a). Further analysis at 18 months, however, indicated improved T2 values suggestive of progressive maturation of the regenerative tissue (Fig. 10b).

### Adverse events

Following initial MSC therapy the patient reported swelling and increased discomfort of the treated knee. The knee effusion persisted for 6 weeks. This was managed conservatively with application of ice, a compression bandage and simple analgesia. Due to this observed reaction the second injection at 6 months was reduced to 50 million MSCs (viability of 95%). The patient reported minor swelling and discomfort for 2 weeks, with no additional management required.

## Discussion

This case study highlights the promise and emerging evidence of mesenchymal stem cell therapy in the treatment of articular pathology including isolated chondral lesions and osteoarthritis. Whilst part of a broader ethics approved and registered case series, this isolated case represents a unique observation due to the underlying pathology of Osteochondritis Dissecans and a history of subsequent failed surgical interventions.

Formal quantitative questionnaire based follow-up indicated consistent improvement in pain and function up to completion of data collection at 18 months. Perhaps the most striking observation was an improvement in Quality of Life (KOOS) by a factor of greater than 400% above pre-treatment baseline. This alone highlights the importance of emerging regenerative techniques.

Other treatment options including biological therapies such as platelet-rich plasma have also noted similar pain and function improvement in patients with symptomatic degenerative knee conditions [54–57]. The disease modification and structural improvement in the underlying articular pathology observed in this case study, however, suggests that MSC therapy may offer significant advantages in regards to changing the end point of joint degeneration – i.e. total joint arthroplasty.

Routine MRI follow-up showed evidence of appreciable improvement in cartilage volume and osteochondral architecture at the site of injury. Early MRI T2 mapping at 6 months indicated that the area of cartilage regeneration exhibited high water content suggestive of immature cartilage or fibrocartilage morphology. Later T2 mapping at 18 months indicated progressive maturation of cartilage from deep to superficial layers with more hyaline like cartilage morphology. Whilst a surgical biopsy would provide true histopathology of the regenerated tissue, a repeat arthroscopy, for purpose of biopsy only, was not felt to be justifiable.

Biological cartilage repair techniques are an emerging area of development with methods such as autologous chondrocyte implantation showing encouraging results [58]. Such techniques however may be limited by donor site morbidity, development of fibrocartilage due to down regulation of chondrocytes during ex vivo expansion and observed poor integration with neighbouring cartilage [59–61]. They are also limited to isolated chondral pathology, and are not transferrable to the more diffuse compartmental changes of osteoarthritis.

In this single case study, the use of isolated expanded adipose derived MSCs, resulted in the regeneration of articular surfaces and subsequent improvement in the patient’s pain and function where previous accepted therapies including autologous chondrocyte implantation had failed. The use of a simple injectable technique with no requirement for invasive intra-articular surgery with its associated risks and complications is an exciting possibility, particularly in the pathology of OA where there exists diffuse rather than isolated cartilage loss.

Whilst the capacity of MSCs to differentiate into chondrocytes has indicated their promise in the management of degenerative chondral pathologies, previous studies have not consistently shown their integration within articular cartilage [62, 63]. This suggests that the benefit of MSC therapy may in fact be achieved through cell to cell contact and paracrine mechanisms (i.e. cytokine expression) leading to a manipulation and improvement of the underlying disease process [32].

Importantly, there remains a number of variables unique to this case study that may be relevant to the observed improvement. The patient was young, had previous surgery which disrupted the osteochondral environment and had a history of OCD which itself may effect outcome and cellular response due to underlying osteochondral pathology. Whether such results would be achieved using a similar intervention in older patients, and with no prior history of OCD, is a question which remains to be answered.

## Conclusion

Osteochondritis Dissecans is an important cause of non-traumatic joint pain in adolescents. Current accepted surgical techniques for isolated osteochondral lesions are limited by factors including technical difficulty, poor tissue integration and development of fibrocartilage. In this case study, mesenchymal stem cell therapy has shown promise in the treatment of osteochondral defects with evidence of structural, functional and pain improvement.

Further research is required to determine if the benefits of mesenchymal stem cell therapy are reproducible across a larger population and for other degenerative joint conditions including osteoarthritis.

## Abbreviations

BSC: Biosafety cabinet
FACS: Florescent Activated Cell Sorting
FBS: Fetal Bovine Serum
ICRS: International Cartilage Repair Society
KOOS: Knee Injury and Osteoarthritis Outcome Score
MRI: Magnetic Resonance Imaging
MSC: Mesenchymal stem cells
NPRS: Numeric pain rating scale)
OCD: Osteochondritis dissecans
PBS: Phosphate buffered saline
WOMAC: Western Ontario and McMaster Universities Arthritis Index
